# Enhancing the Antioxidant Activity of Tea (*Camellia sinensis*) Through Common Herbal Infusions

**DOI:** 10.3390/foods13203284

**Published:** 2024-10-16

**Authors:** Sofia Ortiz-Islas, Claudia A. Espinosa-Leal, Tzitziki González-Rodríguez, Silverio García-Lara

**Affiliations:** 1 Escuela de Ingeniería y Ciencias (EIC), Tecnológico de Monterrey, Eugenio Garza Sada 2501, Monterrey C.P. 64849, Nuevo Leon, Mexico; 2 Department of Chemical Engineering, Massachusetts Institute of Technology (MIT), Cambridge, MA 02139, USA

**Keywords:** tea, infusions, phenolic compounds, antioxidant activity, nutraceuticals

## Abstract

Tea is the second most widely consumed beverage globally, after water, and is known for its substantial antioxidant properties, primarily due to its phenolic content. This study quantifies phenolic compounds and assesses antioxidant activity in ten types of tea and selected herbal infusions, individually and in combination. Our findings reveal that free phenolic compounds and their antioxidant activity were twelve times and eight times greater than bound phenolic compounds. Among individual infusions, white tea exhibited the highest antioxidant activity and phenolic content, with 172.51 µmol TE/1000 g and 7.83 mg GAE/1000 g, respectively. In combination, white/linden flower tea showed the highest antioxidant activity (374.44 µmol TE/1000 g), and white/orange tea contained the highest phenolic content (9.24 mg GAE/1000 g). This study identified primarily two phenolic compounds, epigallocatechin gallate and epicatechin gallate, and one alkaloid, caffeine, in tea and herbal combinations. Compared to other combinations, we observed significant variations in catechins and caffeine between white and dark teas. Integrating specific herbal infusions with tea can enhance antioxidant activity up to three-fold compared to tea alone. This research offers valuable insights into optimizing herbal infusions to maximize antioxidant benefits, creating new opportunities to enhance the health benefits of tea-based products.

## 1. Introduction

An infusion is a process that extracts constituents from plant material, such as leaves, flowers, fruit, or bark, into water. Typically, this is performed by immersing the material in water over time, often with the application of heat to enhance the process [1]. Tea is a specific type of infusion made mainly from leaves of the *Camellia sinensis* plant [2]. Thus, while all teas are infusions, not all infusions are teas. Tea is the second-most consumed liquid in the world after water [3]. Herbal infusions are experiencing growing global popularity, with market projections indicating that sales will reach billions of dollars in the coming years. By 2028, the global herbal tea market is anticipated to reach $4.14 billion [1,4,5].

These beverages are a significant source of bioactive compounds, mainly phenolic acids, which offer numerous health benefits [6]. Their antioxidant activity (AOX) can help alleviate inflammation and prevent obesity, cardiovascular diseases, and certain types of cancer [7,8]. For instance, the polyphenols in black and green tea leaves inhibit lipid intake, combating obesity [9,10]. Green tea can also prevent cardiovascular diseases by reducing oxidative stress, inflammation, proliferation, and platelet aggregation [11,12]. Tea can also improve insulin sensitivity and exhibit antidiabetic properties by decreasing oxidative stress associated with insulin resistance [13].

Tea’s flavor profile is complex and characterized by bitterness, astringency, umami, and sweetness. Bitterness and astringency are primarily due to catechins, flavonol-*O*-glycosides, and tannins [14,15]. Umami is attributed to gallic and succinic acid, L-theanine, and theogallin, while the sweetness in green tea results from the hydrolysis of galloylated catechins [14,16]. These properties are influenced by various factors, including processing methods and agronomic practices [15].

The rising consumption of herbal beverages rich in bioactive compounds reflects growing consumer awareness of their potential health benefits [1,4]. A recent study in Mexico identified chamomile, lemon, peppermint, green and black tea, and linden flowers as the most consumed infusions [17]. However, despite several studies on the composition of these popular infusions, the total phenolic content and AOX of those available in local markets remain underexplored. Furthermore, no studies to date have examined how combining tea with herbal infusions might affect their AOX.

This study aims to investigate how herbal infusions can enhance tea’s AOX. We compared the phenolic content and AOX activity of common infusions and teas, individually and in various combinations. The findings could guide consumers toward the most beneficial infusions in terms of antioxidant activity.

## 2. Materials and Methods

### 2.1. Plant Material

This study evaluated ten commercially available herbal infusions obtained from the local market (Table 1). Five of these infusions were derived from a single species (denoted as I), while the other five were laboratory-prepared combinations of white tea and the most used individual infusions in a 1:1 (*w*/*w*) ratio (denoted as C). Each combination followed a standard preparation of 15 g per tea bag. The extracts were then prepared in triplicate, as described below. Subsequently, all extracts were used for the determination of total phenolics using the Folin–Ciocalteu assay and antioxidant capacity using ORAC, both in triplicate.

### 2.2. Chemical and Reagents

Sodium hydroxide (NaOH, Cat. No. 221465), hydrochloric acid (HCl, Cat. No. 320331), ethyl acetate (Cat. No. 270989), hydrogen peroxide (H_2_O_2_, Cat. No. 216763), methanol (Cat. No. 32221), 2,2′-azo-bis (2-amidinopropane) dihydrochloride (AAPH, Cat. No. 440914), nitrogen gas, hexane, Folin–Ciocalteu reagent, gallic acid, (±)-6-hydroxy-2,5,7,8-tetramethylchromane-2-carboxylic acid (Trolox, Cat. No. 238813), epigallocatechin-3-gallate (95%, HPLC), epicatechin-3-gallate (95%, HPLC), and anhydrous caffeine (99%) were purchased from Sigma-Aldrich (St. Louis, MO, USA). Gibco^®^ phosphate-buffered saline (PBS), pH 7.4 (Cat. No. 10010), was acquired from Thermo Fisher Scientific (Waltham, MA, USA).

### 2.3. Extraction of Free (FPA) and Bound (BPA) Phenolic Acids 

Phenolic acids were extracted using the method described by García-Lara and Bergvinson [17]. One gram of plant material was mixed with 10 mL of 80% methanol (*v*/*v*), shaken at 50 rpm for 10 min, and centrifuged at 2500 rpm and 18 °C for 10 min. The supernatant, containing the FPA, was concentrated to 2 mL at 35 °C and 100 mbar using a vacuum evaporator (Speed VacPlus centrifugal SC250 DDA, Savant, Holbrook, NY, USA). The pellets were digested in 10 mL of 2 M NaOH at 23 °C for one hour under N_2_ gas agitation. After neutralization with 0.5 N HCl, the mixture was extracted with hexane and then five times with ethyl acetate. The combined ethyl acetate fraction was evaporated to dryness and reconstituted in 10 mL of water, representing BPA. Extracts were stored at −20 °C until analysis.

### 2.4. Determination of Total Phenolics by the Folin–Ciocalteu Assay

Total phenolic compounds in each infusion were quantified using the method described by Out et al. [18]. Briefly, 500 μL of FPA or BPA extracts were mixed with 300 μL of 1.5 M H_2_O_2_. The mixture was vortexed and then analyzed using the Folin–Ciocalteu (F–C) assay in a 96-well microplate. Each well contained 15 μL of extract, 240 μL of water, and 15 μL of 0.25 N F–C reagent. After a 3 min incubation, 30 μL of 1 N Na_2_CO_3_ was added, and the mixture was incubated in the dark at 23 °C for 2 h. Absorbance was measured at 765 nm using a microplate reader (Epoch, BioTek Instruments, Inc., Winooski, VT, USA). Phenolic concentrations were expressed as mg of gallic acid equivalents (GAE) per 1000 g dry sample weight.

### 2.5. Antioxidant Capacity Measurement

The hydrophilic antioxidant capacity (AOX) was determined by the oxygen radical absorbance capacity (ORAC) assay for both FPA and BPA extracts. According to Out et al. [18], the extracts were assessed against a Trolox standard with fluorescein as the probe. Peroxyl radicals were generated by 2,2′-azobis(2-amidinopropane) dihydrochloride, and the reduction in fluorescence was monitored using a microplate reader (Synergy™ HT Multi-Detection, BioTek, Inc., Winooski, VT) with excitation and emission wavelengths set at 485 nm and 538 nm, respectively. Results were reported as Trolox equivalents (μmol TE per 1000 g of dry sample weight).

### 2.6. UPLC Analysis

Catechins and caffeine were quantified using a Dionex Ultimate 3000 UPLC system (Thermo Fisher Scientific Inc., USA). The UPLC system was equipped with a Luna 5 µm phenyl–hexyl column (4.6 mm × 250 mm; Phenomenex, Torrance, CA, USA), and the column temperature was maintained at 25 ± 0.5 °C. Detection was performed using a UV–Vis detector with a wavelength set at 278 nm, as outlined by Deka et al. [19]. 

### 2.7. Statistical Analysis

Ten different infusions were analyzed in triplicate across two separate experiments. Data are presented as mean ± standard error of the mean. Statistical significance was determined using one-way ANOVA followed by Tukey’s test, with a significance threshold of *p* < 0.05. This analysis was applied to FPA and BPA, total phenolic compounds, and antioxidant activity (AOX). Comparisons were made based on extraction methods (methanol vs. boiling water), FPA and BPA content across different infusions, and the effect of individual species versus their combinations. All statistical analyses were performed using RStudio software version 2024.04.2.

## 3. Results

### 3.1. Comparison of Extraction Methods for FPA and BPA

**Figure 1:**
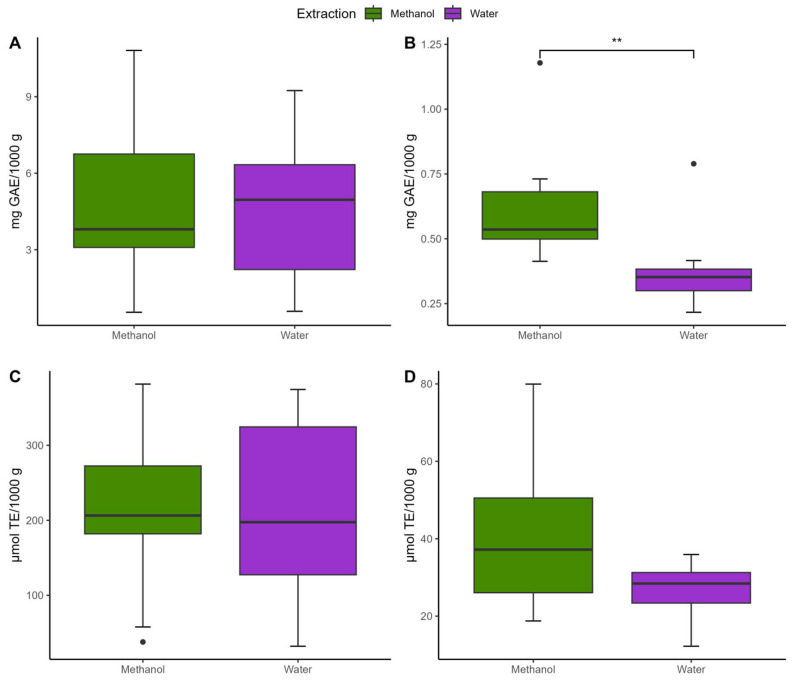
Comparison of phenolic compound extraction using the standardized method (methanol) and the conventional method (water). The comparison includes both herbal infusions and plant teas. (**A**) Total free phenolic compounds; (**B**) Total bound phenolic compounds; (**C**) Antioxidant activity of free phenolic compounds; (**D**) Antioxidant activity of bound phenolic compounds. * Indicates statistically significant difference (n = 30; ** *p* < 0.01). No other statistically significant differences were detected between the two extraction methods for any of the remaining parameters tested.

In the initial phase of this study, we conducted a comparative analysis of two extraction methods (methanol and water) using ten different infusions (Table 1). Figure 1 shows the results of this comparison. On average, the methanolic extraction yielded 4.96 mg GAE/1000 g of FPA, while the water extraction resulted in 4.63 mg GAE/1000 g (Figure 1A). For BPA, methanol extraction produced 0.62 mg GAE/1000 g, whereas water extraction resulted in 0.38 mg GAE/1000 g (Figure 1B). Regarding antioxidant activity, methanolic extraction yielded 209.55 µmol TE/1000 g for FPA and 212.43 µmol TE/1000 g with water (Figure 1C). Conversely, the AOX for BPA was 40.03 µmol TE/1000 g with methanol and 26.52 µmol TE/1000 g with water (Figure 1D). Statistically significant differences were observed only for total BPA acids between the two methods. Since water is environmentally friendly, cost-effective, and aligns with typical tea brewing methods, it was selected as the extraction solvent for all subsequent analyses.

**Figure 2:**
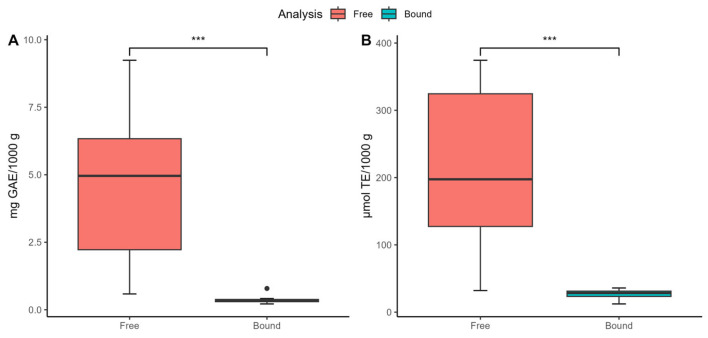
Comparative analysis of free and bound phenolic compounds across herbal infusions and teas. (**A**) Quantitative content. (**B**) Antioxidant activity. * Indicates statistically significant difference (n = 30; *** *p* < 0.001).

Figure 2 compares the quantity and antioxidant activity of phenolic compounds extracted with water, distinguishing between FPA and BPA. The results reveal that FPA significantly surpasses BPA in both content and antioxidant activity. Specifically, FPA contains 4.63 mg of gallic acid equivalents (GAE) compared to 0.38 mg for BPA (Figure 2A) and exhibits an AOX of 212.43 µmol TE versus 26.51 µmol TE for BPA (Figure 2B). This indicates that free phenolic compounds are approximately 12 times more abundant and eight times more active in AOX than bound phenolic compounds. These findings suggest that FPA is the primary source of phenolics and their antioxidant activity in tea and herbal infusions.

### 3.2. FPA, BPS, and AOX in Herbal Infusions and Teas

**Figure 3:**
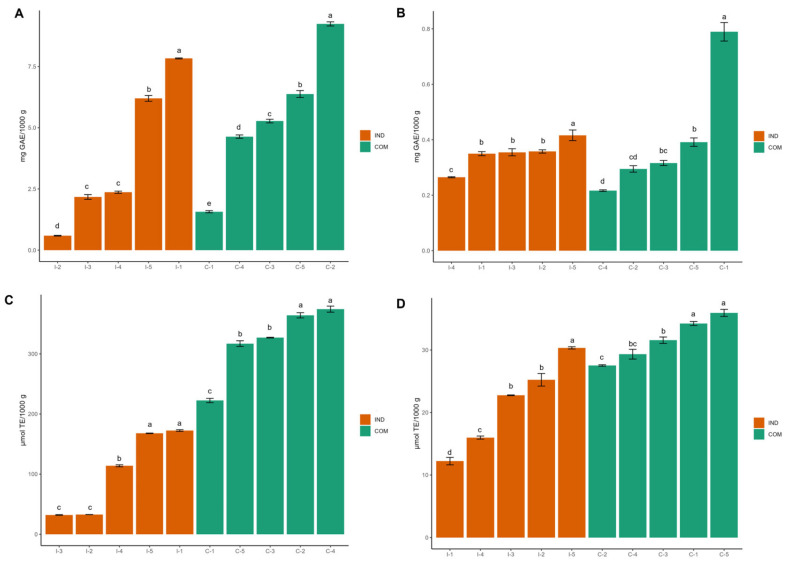
Comparative analysis of free phenolic compound content and antioxidant activity (AOX): (**A**) Total free phenolic compounds; (**B**) Total bound phenolic compounds; (**C**) AOX of free phenolic compounds; and (**D**) AOX of bound phenolic compounds across individual herbal infusions and teas, as well as their combinations. Distinct letters represent statistically significant differences (n = 3; *p* < 0.05). Group-ID: I-1: *Camellia sinensis*; I-2: *Citrus aurantium*; I-3: *Cinnamomum zeylanicum*; I-4: *Mentha piperita*; I-5: *Ternstroemia pringlei*; C-1: *Camellia sinensis*/*Cinnamomum zeylanicum*; C-2: *Camellia sinensis*/*Citrus aurantium*; C-3: *Camellia sinensis*/*Mentha piperita*; C-4: *Camellia sinensis*/*Ternstroemia pringlei*; C-5: *Camellia sinensis* (Var white)/*Camellia sinensis* (Var black).

Based on the results obtained, specific infusions commonly available in the local market were analyzed. Figure 3 compares the content of phenolic compounds and antioxidant activity between individual and combined infusions. As expected, significant variations were observed in both FPA and BPA content and AOX across different infusions. Notably, there were also marked differences between combined infusions regarding FPA, BPA, and AOX. 

For FPA, the highest individual content was 7.83 mg GAE/1000 g in white tea (I-1), whereas the most effective combination was in white/orange tea (C-2), with 9.24 mg GAE/1000 g (Figure 3A). For BPA, linden flower tea (I-5) had the highest individual content (0.41 mg GAE/1000 g), while the optimal combined preparation was white/cinnamon tea (C-1), which had 0.78 mg GAE/1000 g (Figure 3B).

Remarkably, white tea (I-1) exhibited the highest AOX from FPA (172.51 µmol TE/1000 g), closely followed by linden flower tea (I-5) which exhibits 167.92 µmol TE/1000 g. The most effective combinations for AOX included white/linden flower tea (C-4) with 374.44 µmol TE/1000 g and white/orange tea (C-2) with 364.13 µmol TE/1000 g (Figure 3C). For BPA, linden flower tea (I-5) showed the highest AOX as an individual infusion (30.34 µmol TE/1000 g). However, the most potent combinations were found in white/black tea (C-5) with 35.94 µmol TE/1000 g and white/cinnamon tea (C-1) with 34.25 µmol TE/1000 g (Figure 3D). 

### 3.3. Comparison between Individual and Combined Infusions

**Figure 4:**
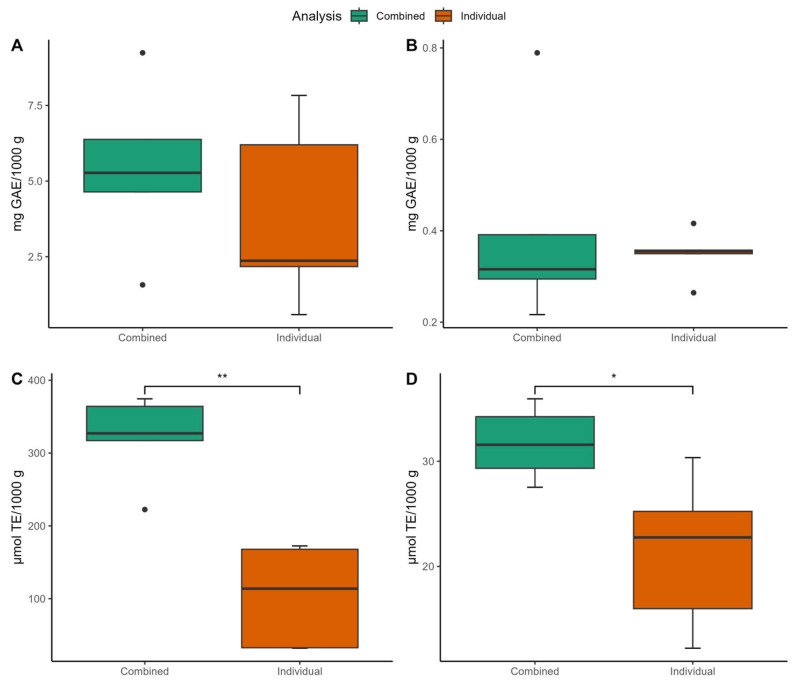
Comparative analysis of herbal infusion preparations. (**A**) Total free phenolic compounds; (**B**) Total bound phenolic compounds; (**C**) AOX of free phenolic compounds; and (**D**) AOX of bound phenolic compounds when using individual species or a combination. * Indicates statistically significant difference (n = 15; * *p* < 0.05; ** *p* < 0.01).

The utilization of individual herbal infusions and their combinations for preparation was analyzed, as illustrated in Figure 4. The content of FPA for individual species was 3.83 mg GAE/1000 g while, after a specific combination, it increased to 5.43 mg GAE/1000 g (Figure 4A). A similar pattern was observed for BPA, with an increase from 0.35 mg GAE/1000 g in individual tea to 0.40 mg GAE/1000 g in the combinations (Figure 4B). However, the content of FPA and BPA did not show significant differences between individual and combined preparation methods. 

A similar trend was observed for the AOX of FPA, which significantly differed between individual and combined herbal preparations. The activity for individual species was lower (103.82 µmol TE/1000 g) compared to the combination (321.04 µmol TE/1000 g) (Figure 4C). For the AOX of the BPA, the µmol TE/1000 g increased from 21.31 to 31.72 on average (Figure 4D).

This indicates that combining tea with specific herbal infusions enhanced the antioxidant activity of the final product up to three times compared to the original activity. This enhancement is primarily attributed to the presence of free phenolic compounds in both the tea and herbal species used in this study.

### 3.4. Main Compounds in Combined Infusions

Table 2 presents the phenolic compounds (catechins) and alkaloids in extracts from *Camellia sinensis* combined with herbal teas quantified by UPLC. The primary phenolic compounds detected were epigallocatechin gallate and epicatechin gallate, along with the alkaloid caffeine. Among these, epigallocatechin gallate had the highest concentration, followed by caffeine and epicatechin gallate. The content of epigallocatechin gallate ranged from 167 to 215 mg/g; caffeine ranged from 43 to 52 mg/g, and epicatechin gallate ranged from 36 to 49 mg/g. Significant differences were noted between combinations C-4 and C-5 compared to C-1, C-2, and C-3. Notably, combinations C-4 and C-5 exhibited approximately a 1.2-fold increase in content compared to the other combinations. The main compound concentrations did not show a dependence on the type of combination, except when *Camellia sinensis* was combined with itself (white and black variety) or with linden flower tea.

## 4. Discussion

The extraction of phenolic compounds using the conventional method (water) yielded quantities comparable to those obtained with methanol. This result was anticipated, as water primarily extracts FPA [20]. However, studies such as Guimarães et al. [21] have reported lower phenolic levels from *Matricaria recutita* using a common infusion compared to methanol extraction. Therefore, we chose water as a solvent to extract soluble phenolic compounds in this study, as it facilitates better comparisons across infusions and is a universal and human-compatible solvent. As expected, tea and herbal infusions have a higher concentration of FPA than BPA [22]. Additionally, it would have been beneficial to evaluate different brewing [2,3,23] and preparation methods [24], as evidence suggests that these factors significantly impact compound content, such as the higher transfer of minerals and polyphenols observed with the filter infusion method [24].

Our study found that using plant species individually or in combination significantly influences the content of soluble phenolic compounds and the antioxidant activity associated with this fraction in an infusion or tea. Similar findings were reported by Apak et al. [22], who found that different blends affected the AOX of herbal infusions, with green tea exhibiting higher AOX than a blend of black tea. Our results also suggest that the quantities of phenolic compounds vary across plant species used to produce the infusion. The association between phenolic compound content and AOX of herbal infusions and tea was not observed in this study. This can be explained by the unspecific nature of the method employed to determine antioxidant activity, as it can react to other compounds that are not phenols [19]. 

Specific herbal infusions, such as linden flower, exhibited higher total phenolic compounds (catechins), while others, including orange and chamomile, had lower quantities than tea infusions. Atoui et al. [6] discovered that linden flower had a higher phenolic content than other herbal infusions, including chamomile, but black and green teas were superior. Diminš and Augšpole et al. [25] reported similar findings, where linden flower infusion had a higher phenolic compound content than chamomile infusion. In contrast to our study, Karakaya and Nehir [26] found that linden flower infusion had a lower AOX than black tea. 

Tea has a high polyphenolic compound content, especially in green and black varieties and linden flowers. Linden flowers, in particular, have a high concentration of catechins [6] and flavonoids. The antioxidant activity is related to the molecule’s position and degree of hydroxylation [26]; thus, the extraction process may alter molecules and affect the resulting AOX. Other factors, such as the origin of the plant [27,28], species diversity, and overall processing [27], can influence the concentration of phenolic compounds and AOX found in the infusions.

The total phenolic content and its corresponding AOX activity among the tea infusions tested in our study align with the findings of Quesille-Villalobos et al. [27]. They found that white and green tea infusions exhibited higher total phenolic contents. The processing of the leaves, such as the fermentation of black tea, influences the levels of phenolic compounds. For instance, the oxidation of catechins, commonly found at high levels in non-fermented teas, during the fermentation process increases the number of phenolic molecules, such as theaflavins and thearubigins in black teas [27,28], thereby reducing AOX.

This result clarifies that the antioxidant effects were promoted not only by the phenolic contents present in white tea (catechins and caffeine) or linden flower tea (catechins) but also by different phenolic compounds produced from herbal extraction, which agrees with previous studies [18,24,28]. Although caffeine is one of the three main alkaloid compounds in white tea leaves, it did not show antioxidant effects in earlier studies [19].

Our study underscores the practical implications of understanding the phenolic content and AOX of different infusions. This knowledge can guide consumers in their choice of beverage by providing information about the AOX activity of the available infusions. It can also assist researchers and producers in selecting and enhancing commercially available infusions. Among the herbal species tested, white tea exhibited superior free phenolic content and AOX, followed by linden flowers. 

The global tea industry continues to evolve, reflecting its historical significance and contemporary relevance [1]. Given its high health potential [29], integrating scientific research with industry practices is crucial to maximizing the health benefits of tea and herbal infusions. This approach will help ensure that both consumers and the industry benefit from the latest advancements and evidence-based insights.

## 5. Conclusions

This study underscores the potential of infusions and tea as rich sources of antioxidants and affordable nutraceuticals. White tea exhibited the highest antioxidant activity among individual infusions, while linden flower infusion had the highest phenolic content. The white/orange tea blend demonstrated the highest antioxidant activity among specific combinations, whereas the white/linden flower tea blend had the highest phenolic content. Notably, the tea and herbal combinations identified two phenolic compounds (epigallocatechin gallate and epicatechin gallate) and one alkaloid (caffeine), providing insights into their composition. Combining tea with specific herbal infusions enhanced the antioxidant activity of the final product by up to three times compared to the original activity. These findings highlight the potential for further research to identify the compounds contributing to the antioxidant activity in all tea varieties, thereby empowering the industry and consumers with valuable information.

## Figures and Tables

**Table 1:** List of the herbal infusions and teas analyzed in this study, all available in the local market. Groups: “Individual” (I) or “Combined” (C). The “ID” column refers to the unique identification number for each infusion or tea.

Group-ID	Species	Tissue	Common Name	Brand/Batch
I-1	*Camellia sinensis*	Leaves, Flowers	White Tea	McCormick, Mexico, 09-2022
I-2	*Citrus aurantium*	Leaves, Flowers	Orange Tea	Lagg’s, Mexico, 10-2022
I-3	*Cinnamomum zeylanicum*	Cortex	Cinnamon Tea	Lagg’s, Mexico, 10-2022
I-4	*Mentha piperita*	Leaves	Peppermint Tea	Lagg’s, Mexico, 10-2022
I-5	*Ternstroemia pringlei*	Flowers	Linden Flowers Tea	La Pastora, Mexico, 09-2022
C-1	*Camellia sinensis* *Cinnamomum zeylanicum*,	1:1 Leaves and Cortex	White Tea with Cinnamon Tea	McCormick, Mexico, 09-2022
C-2	*Camellia sinensis*, *Citrus aurantium*	1:1 Leaves	White Tea with Orange Tea	Lagg’s, Mexico, 10-2022
C-3	*Camellia sinensis*, *Mentha piperita*	1:1 Leaves	Green Tea with Peppermint Tea	Lagg’s, Mexico, 10-2022
C-4	*Camellia sinensis* *Ternstroemia pringlei*	1:1 Leaves, Flowers	White Tea with Linden Flowers Tea	Lagg’s, Mexico, 10-2022
C-5	*Camellia sinensis* (Var white), *Camellia sinensis* (Var black)	1:1 Leaves, Flowers	White Tea with Black Tea	McCormick, Mexico, 09-2022

**Table 2:** Content of major catechins and caffeine for *Camellia sinensis* in combination with herbal extracts.

Tea Combination	Caffeine (mg g^−1^)	Epigallocatechin Gallate (mg g^−1^)	Epicatechin Gallate (mg g^−1^)
C-1	44.50 ± 0.21 a	168.24 ± 2.77 a	36.44 ± 1.14 a
C-2	43.91 ± 0.49 a	167.06 ± 1.24 a	36.00 ± 0.55 a
C-3	44.01 ± 0.73 a	169.40 ± 1.94 a	36.52 ± 0.89 a
C-4	49.13 ± 0.60 b	196.23 ± 1.40 b	46.21 ± 0.55 b
C-5	52.47 ± 0.78 b	215.98 ± 2.14 b	49.02 ± 1.04 b

The data are expressed as mean values (± S.D.) from three experiments. Different letters denote statistically significant differences (*p* < 0.05). Group-ID: C-1: *Camellia sinensis*/*Cinnamomum zeylanicum*; C-2: *Camellia sinensis*/*Citrus aurantium*; C-3: *Camellia sinensis*/*Mentha piperita*; C-4: *Camellia sinensis*/*Ternstroemia pringlei*; C-5: *Camellia sinensis* (Var white)/*Camellia sinensis* (Var black).

## Data Availability

The data presented in this study are available on request from the corresponding author.

## References

[1] Huda H., Majid N.B.A., Chen Y., Adnan M., Ashraf S.A., Roszko M., Bryła M., Kieliszek M., Sasidharan S. (2024). Exploring the Ancient Roots and Modern Global Brews of Tea and Herbal Beverages: A Comprehensive Review of Origins, Types, Health Benefits, Market Dynamics, and Future Trends. Food Sci. Nutr..

[2] Winiarska-Mieczan A., Baranowska-Wójcik E. (2024). The Effect of Brewing Time on the Antioxidant Activity of Tea Infusions. Appl. Sci..

[3] Aydemir M.E., Takım K., Yılmaz M.A. (2024). Characterization of Phenolic Components of Black Teas of Different Origins and the Effect of Brewing Duration on Quality Properties. Food Sci. Nutr..

[4] Sousa A.C., Pádua I., Gonçalves V.M.F., Ribeiro C., Leal S. (2024). Exploring Tea and Herbal Infusions Consumption Patterns and Behaviours: The Case of Portuguese Consumers. Heliyon.

[5] Shaik M.I., Hamdi I.H., Sarbon N.M. (2023). A Comprehensive Review on Traditional Herbal Drinks: Physicochemical, Phytochemicals and Pharmacology Properties. Food Chem. Adv..

[6] Atoui A.K., Mansouri A., Boskou G.P. (2005). Tea and herbal infusions: Their antioxidant activity and phenolic profile. Food Chem..

[7] Chaudhary P., Mitra D., Das Mohapatra P.K., Oana Docea A., Mon Myo E., Janmeda P., Martorell M., Iriti M., Ibrayeva M., Sharifi-Rad J. (2023). *Camellia sinensis*: Insights on Its Molecular Mechanisms of Action towards Nutraceutical, Anticancer Potential and Other Therapeutic Applications. Arab. J. Chem..

[8] Sanlier N., Gokcen B.B., Altuğ M. (2018). Tea consumption and disease correlations. Trends Food Sci. Technol..

[9] Xu X.-Y., Zhao C.-N., Li B.-Y., Tang G.-Y., Shang A., Gan R.-Y., Feng Y.-B., Li H.-B. (2023). Effects and Mechanisms of Tea on Obesity. Crit. Rev. Food Sci. Nutr..

[10] Xiao T., Li Y., Li H., Wang K., Huang J., Liu Z., Zhu M. (2024). Tea Consumption in Relation with Metabolic Syndrome and Obesity: A Systematic Review and Meta-Analysis of Randomized Clinical Trials. Food Biosci..

[11] Guo J., Li K., Lin Y., Liu Y. (2023). Protective Effects and Molecular Mechanisms of Tea Polyphenols on Cardiovascular Diseases. Front. Nutr..

[12] Zamani M., Kelishadi M.R., Ashtary-Larky D., Amirani N., Goudarzi K., Torki I.A., Bagheri R., Ghanavati M., Asbaghi O. (2023). The Effects of Green Tea Supplementation on Cardiovascular Risk Factors: A Systematic Review and Meta-Analysis. Front. Nutr..

[13] Wei Y., Shao J., Pang Y., Wen C., Wei K., Peng L., Wang Y., Wei X. (2024). Antidiabetic Potential of Tea and Its Active Compounds: From Molecular Mechanism to Clinical Evidence. J. Agric. Food Chem..

[14] Zhang L., Cao Q.-Q., Granato D., Xu Y.-Q., Ho C.-T. (2020). Association between Chemistry and Taste of Tea: A Review. Trends Food Sci. Technol..

[15] Ye J.-H., Ye Y., Yin J.-F., Jin J., Liang Y.-R., Liu R.-Y., Tang P., Xu Y.-Q. (2022). Bitterness and Astringency of Tea Leaves and Products: Formation Mechanism and Reducing Strategies. Trends Food Sci. Technol..

[16] Chen Y.-H., Zhang Y.-H., Chen G.-S., Yin J.-F., Chen J.-X., Wang F., Xu Y.-Q. (2022). Effects of Phenolic Acids and Quercetin-3-O-Rutinoside on the Bitterness and Astringency of Green Tea Infusion. Npj Sci. Food.

[17] The Food Tech. https://thefoodtech.com/tendencias-de-consumo/conoce-las-tendencias-en-alimentos-y-bebidas-2024-de-mintel-para-latam/.

[18] Ou B., Hampsch-Woodill M., Prior R.L. (2001). Development and validation of an improved oxygen radical absorbance capacity assay using fluorescein as the fluorescent probe. J. Agric. Food Chem..

[19] Deka H., Sarmah P.P., Devi A., Tamuly P., Karak T. (2021). Changes in major catechins, caffeine, and antioxidant activity during CTC processing of black tea from North East India. RSC Adv..

[20] Su D., Zhang R., Hou F., Zhang M., Guo J., Huang F., Deng Y., Wei Z. (2014). Comparison of the free and bound phenolic profiles and cellular antioxidant activities of litchi pulp extracts from different solvents. BMC Complement. Altern. Med..

[21] Guimarães R., Barros L., Dueñas M., Calhelha R.C., Carvalho A.M., Santos-Buelga C., Queiroz M.J., Ferreira I.C. (2013). Infusion and decoction of wild German chamomile: Bioactivity and characterization of organic acids and phenolic compounds. Food Chem..

[22] Apak R., Güçlü K., Ozyürek M., Esin Karademir S.E., Erçag E. (2006). The cupric ion reducing antioxidant capacity and polyphenolic content of some herbal teas. Int. J. Food Sci. Nutr..

[23] Długaszek M., Mierczyk J. (2024). Elemental Composition of Green Tea Infusions Depending on the Method of Their Brewing. Eur. Food Res. Technol..

[24] Lo Turco V., Nava V., Potortì A.G., Sgrò B., Arrigo M.A., Di Bella G. (2024). Total Polyphenol Contents and Mineral Profiles in Commercial Wellness Herbal Infusions: Evaluation of the Differences between Two Preparation Methods. Foods.

[25] Dimiņš F., Augšpole I. (2019). Key engineering materials. Total Phenolic, Antioxidant Activities, and Flavonoid Contents of Herbal Syrups.

[26] Karakay S., El S., El S.N. (2006). Total phenols and antioxidant activities of some herbal teas and in vitro bioavailability of black tea polyphenols. Ziraat Fak. Derg..

[27] Quesille-Villalobos A.M., Torrico J.S., Ranilla L.G. (2013). Phenolic compounds, antioxidant capacity, and in vitro α-amylase inhibitory potential of tea infusions (*Camellia sinensis*) commercialized in Chile. J. Food.

[28] Rusak G., Komes D., Likić S., Horžić D., Kovač M. (2008). Phenolic content and antioxidative capacity of green and white tea extracts depending on extraction conditions and the solvent used. Food Chem..

[29] Luo Q., Luo L., Zhao J., Wang Y., Luo H. (2023). Biological Potential and Mechanisms of Tea’s Bioactive Compounds: An Updated Review. J. Adv. Res..

